# Health-promoting lifestyle and its predictors among health-related and non-health-related university students in Taiwan: a cross-sectional quantitative study

**DOI:** 10.1186/s12889-023-15760-2

**Published:** 2023-05-05

**Authors:** Dan-Ping Chao

**Affiliations:** Department of Tourism and Leisure Management, China University of Technology, No. 56, Sec. 3, Xinglong Rd., Wunshan Dist., Taipei, Taiwan

**Keywords:** Professional fields, Health-promoting behaviors, Gender, Perceived health status, Health conception, Physical activity duration, Disposable income, Dine-outs number

## Abstract

**Background:**

University students majoring in different disciplines are believed to have different personality traits, courses exposure, and future roles, which may further affect their health behaviors and health status. The purpose of this study was to investigate the differences in health-promoting lifestyle (HPL) and its predictors among health-related and non-health-related students.

**Methods:**

The research participants were university students in the main island of Taiwan, and a two-stage sampling approach was adopted to obtain the samples from November 2020 to March 2021. First, 37 universities were randomly selected based on the ratio of public and private universities in each region of Taiwan. Then, based on the ratio of health-related and non-health-related majors of selected university, 25–30 students were randomly drawn from each university according to the student ID number to complete self-administered questionnaires, which included items for personal factors, perceived health status (PHS), health conception (HC), and health-promoting lifestyle profile (HPLP). A total of 1062 valid questionnaires were recovered, including 458 from health-related students and 604 from non-health-related students. Chi-squared test, independent samples t-test, one-way ANOVA, Pearson product-moment correlation analysis, and multiple regression analysis were performed.

**Results:**

The results showed that gender (*p* < 0.001), residential status (*p* = 0.023), body mass index (*p* = 0.016), and daily sleep duration (*p* = 0.034) of the students majoring in different disciplines were different. Health-related students having better HC (*p* = 0.002) and HPLP (*p* = 0.040) than non-health-related students. In addition, for both majors, females, low PHS scores, and low scores for functional/role, clinical, and eudaimonistic dimensions of HC were important indicators of a relatively negative HPLP, while health-related students who exercised 75 min or less per week and non-health-related students with a monthly disposable income of 15,000 TWD or less or who dined out 15 times or more per week also required attention in the promotion of HPL (health-related majors: adjusted R^2^ = 0.481, *p* < 0.001; non-health-related majors: adjusted R^2^ = 0.443, *p* < 0.001).

**Conclusions:**

Students majoring in each discipline who had poor HPLP which is mentioned above should be prioritized in the provision of appropriate exercise or nutritional support programs on campus to promote their awareness and ability to pay attention to their health.

**Supplementary Information:**

The online version contains supplementary material available at 10.1186/s12889-023-15760-2.

## Background

In the past 20 years, non-communicable chronic diseases have replaced infectious diseases as important threats to human life, along with even more disabilities [1, 2]. A gradual decline was observed for the age group suffering from chronic diseases. Abnormal metabolism or early diagnosis of chronic diseases have also been observed in younger age groups [3]. Chronic diseases are known to be caused by long-term inappropriate habits and health behaviors [4]. Studies have shown that current dietary behaviors and sedentary lifestyles are likely to lead to obesity, which indirectly increases the incidence and mortality of chronic diseases [5]. Another longitudinal study in the United States also found that respondents who had lower-risk lifestyles, such as non-smoking, having a healthy weight, adequate physical activity, moderate alcohol consumption, and a balanced diet, live longer [6]. It can be observed that chronic diseases have become the key to disease prevention and control nowadays. The healthy lifespan of individuals can only be extended by guiding healthy behaviors through health education and enabling people to develop a healthy lifestyle.

University is one of the critical periods in life when students usually face many challenges, including staying away from family and friends, developing new social networks, adapting to new learning environments and schedules, as well as having more autonomy over their behaviors [7, 8]. Meanwhile, the behaviors of university students are highly likely to change due to the influence of their peers or the environment [9]. In addition, university students typically have just entered adulthood from adolescence and are often still relatively young and healthy, making it more likely for them to ignore their health [10]. Therefore, several studies have shown that university students are more prone to behaviors that endanger their health, such as lack of physical activity, unhealthy eating habits, alcohol consumption, and smoking [7, 11, 12]. Since it is difficult to change a fixed lifestyle after adulthood and university is considered to be the last opportunity for behavioral development and learning, a healthy lifestyle developed at this time may lay the foundation for the health status after middle age to reduce suboptimal health or delay the onset of chronic diseases [10, 13]. Therefore, it is imperative to consider in the current situation and educational needs of health-promoting behaviors of university students and to plan appropriate health education programs for the correct target audience.

Health-promoting lifestyle (HPL) may be regarded as a positive way of life, which consists of various health-promoting behaviors such as self-actualization, health responsibility, exercise, nutrition, interpersonal support, and stress management [14, 15]. Studies from Taiwan and several countries have shown that university students generally have favorable self-actualization and interpersonal support, while improvement is required for stress management, nutrition, exercise, and health responsibility [8, 11, 16–20]. The influencing factors of HPL may be divided into cognitive-perceptual factors and modifying factors. Among them, the cognitive-perceptual factors refer to the main motivations for adopting or maintaining HPLs, including health conception (HC) and perceived health status (PHS). Modifying factors indirectly affect HPLs by affecting cognitive-perceptual factors, including demographic characteristics and behavioral factors. A study showed that HC, PHS, gender, and time spent searching online for health-related information per week may predict the HPL of nursing students in three universities in South Korea [21]. Taiwanese research has also shown that students who had better PHS, had studied healthcare courses, and often attended morning classes tended to have better HPLs in six universities in Taichung [17]. Another study in Taiwan showed that an exercise frequency of three times or more a week is a predictor of HPL in university freshmen [22].

The educational goals and curriculum planning of various majors in universities must certainly correspond to the needs of students and their future roles, and may affect their HPLs. Studies have found that medical students who regularly receive medical and health information have better e-health literacy in Taiwan and Japan [23, 24]. Another study in Taiwan reported that healthy-lifestyle-promoting program in nursing courses improves exercise and nutrition in nursing students [25]. There are also differences in HPLs between students in different disciplines. Some studies have shown that students from medical schools or health-related majors have better overall HPL or health-promoting behaviors such as exercise, nutrition, and health responsibility [7, 8, 10, 19]. Some studies suggested that students with non-medical-related majors had better HPLs [12]. Although several scholars believe that the student’s major may be regarded as one of the predictors of HPL [7, 12, 18, 23], the impact of a major has remained inconclusive. Seldom relevant research in disciplines and HPLs in Taiwan. Moreover, few studies have separately explored the influencing factors of HPL and the direction of health education for different majors to date.

Therefore, the main island of Taiwan was used as the scope of this study to first divide students in various regions and types of universities based on health-related or non-health-related majors to investigate the differences in socio-demographic characteristics, health-related information, PHS, HC, and HPL between university students with different majors. The relationships between cognitive-perceptual factors (PHS and HC), modifying factors (socio-demographic characteristics and health-related information), and the HPL of students were explored to identify the influencing factors likely predictive of the HPL of students in health-related majors and non-health-related majors. The target group with poor health behaviors that required urgent prioritized intervention was specifically identified to propose on-campus health promotion education advice that varied by professional area and met the demands.

## Methods

### Participants

The Ministry of Education (MOE) of Taiwan divides universities into 27 discipline clusters according to the theoretical and practical learning contents of different majors [26]. In this study, university students were divided based on health-related and non-health-related majors using the discipline classification by the MOE to explore the differences in HPL among students majoring in different disciplines. Health-related majors included four disciplines that were directly or indirectly related to human health, such as medicine and health, hospitality/tourism/personal service, life sciences, as well as agriculture. On the other hand, 23 disciplines were classified as non-health-related majors, including education, arts, humanities, languages and literatures, social and behavioral sciences, journalism and library information, business and administration, law, environment, physics/chemistry/earth sciences, mathematics and statistics, information and communication technologies, engineering and engineering trades, manufacturing and processing, architecture and construction, forestry, fisheries, veterinary medicine, social welfare, hygiene and occupational health services, security services, transport services, and field unknown. The purpose, process, benefits, and potential risks of the study were explained by the interviewer in detail to all the university student participants, and the participants filled in the questionnaire after signing the informed consent. A total of 1076 questionnaires were distributed; 1062 valid questionnaires were finally recovered, with an effective rate of 98.7%.

### Study design

This cross-sectional quantitative study was reviewed and approved by the Behavioral and Social Science Research Ethics Committee of National Taiwan University (202004ES028). The ethic code of the *Declaration of Helsinki* was complied with throughout the study. A two-stage sampling approach was used from November 2020 to March 2021. During the first stage, 37 universities were randomly selected from 140 universities based on the ratios of public and private universities in the northern, central, southern, and eastern regions of Taiwan. The second stage, based on the ratio of health-related and non-health-related majors of selected university, 25 to 30 students were randomly drawn from each test university according to the student ID number. If the selected students were not on campus or did not agree to take the test, samples were drawn again as substitutes. During the test, the interviewer delivered the questionnaire, and the participants completed the self-administered questionnaire.

### Measurements

In this study, a questionnaire of closed-ended questions was developed by referencing recent related studies [27–30]. The questionnaire included personal factors (socio-demographic characteristics and health-related information), PHS, HC, and HPL. The socio-demographic characteristics included four variables, namely gender, grade, residential status, and monthly disposable income. The health-related information included six variables, namely body mass index (BMI), daily duration of sleep, daily duration of 3C (computer, communication, and consumer electronics) usage, weekly duration of physical activity, weekly number of dine-outs, and weekly number of night snacks.

Modern research was referenced when developing the PHS scale in this study, which included four items, namely overall self-assessment, health status compared with those of peers, personal health status compared with that six months ago, and health status compared with ideal well-being [27]. Expert review revealed that the content validity was good (mean item-content validity index = 1). A five-point Likert scale was used for scoring, and 1 to 5 points were assigned to responses from “very poor” to “very good,” respectively. A higher mean score indicated a better subjective evaluation of health by the individual. The Cronbach’s alpha and test-retest correlation coefficient of this scale was 0.90 and 0.77, respectively, indicating good internal and external consistency reliability.

The Laffrey Health Conception Scale that was translated into Chinese and simplified into a 24-item version [28, 29] was adopted in this study, which was verified by factor analysis to include the following four dimensions of HC: functional/role performance (7 items), adaptive (7 items), clinical (5 items), and eudaimonistic (5 items). The items were scored on a five-point Likert scale, and 1 to 5 points were assigned to responses ranging from “strongly disagree” to “strongly agree,” respectively. A higher mean score indicated a more positive subjective perception of health by the individual. The Cronbach’s alpha and test-retest correlation coefficient of this scale was 0.94 and 0.90, respectively, which also indicated good reliability.

The original Health-Promoting Lifestyle Profile (HPLP) included six dimensions and a total of 48 items [15]. In this study, the version translated into Chinese and simplified to 24 items by modern scholars was adopted [29, 30]. The questionnaire had been verified by factor analysis that it still contained six dimensions, namely self-actualization, health responsibility, exercise, nutrition, interpersonal support, and stress management. Each dimension included four questions, and the questions were scored on a five-point Likert scale. Scores of 1 to 5 were assigned to responses ranging from “Never” to “Always,” with a higher mean score indicating a lifestyle that was closer to an ideal HPL. The profile also had good reliability (Cronbach’s α = 0.93, test-retest correlation coefficient = 0.87).

### Data analysis

SPSS version 23.0 was used for analysis in this study. The chi-squared test or the independent samples t-test was performed to analyze the differences in personal factors, PHS, HC, and HPLP related to the different majors. The independent samples t-test or one-way ANOVA combined with Scheffé post-hoc test was used to identify the relationship between personal factors and PHS, HC, and HPLP in health-related and non-health-related majors. The Pearson product-moment correlation analysis was performed to determine the correlations between PHS, HC, and HPLP among students with different majors. Moreover, multiple regression analysis was used to identify the predictors of HPLP of students with health-related and non-health-related majors. Regression analysis was performed for all samples and samples by major, respectively, with the HPLP score as the dependent variable. The ten original variables of personal factors were converted into 18 dummy variables, and the scores for PHS and each dimension of HC were added, and a total of 23 independent variables to perform a stepwise regression analysis with an inclusion criterion of 0.05 and an exclusion criterion of 0.10. A *p*-value of < 0.05 indicated statistical significance. Tolerance > 0.1 and variance inflation factor (VIF) < 10 indicated an absence of collinearity between independent variables.

## Results

### Personal factors of different majors

Of the 1062 respondents to the valid questionnaires recovered in this study, 458 studied health-related majors, which accounted for 43.1%, including 252 (23.7%) majoring in the discipline cluster of medicine and health, 159 (15.0%) majoring in hospitality/tourism/personal service, 43 (4.0%) majoring in life sciences, and 4 (0.4%) majoring in agriculture. There were 604 participants with non-health-related majors, which accounted for 56.9%. Please refer to Table 1 for the distribution of socio-demographic characteristics of university students by major. Chi-squared analysis showed significant differences in gender (χ^2^ = 31.808, *p* ˂ 0.001) and residential status (χ^2^ = 7.519, *p* = 0.023) among different majors. More female students had health-related majors (67.9%) than non-health-related majors (50.7%). More health-related students (35.8%) than non-health-related students (29.3%) rented apartments outside the campus, while more non-health-related students were living in school dormitories (34.8%) than health-related students (27.7%).

**Table 1:** Socio-demographic characteristics of university students by major

Variables	Health-related majors (*n* = 458)		Non-health-related majors (*n* = 604)		*p*-value^a^
	*N*	%	*N*	%	
Gender					<0.001^***^
Male	147	32.1	298	49.3	
Female	311	67.9	306	50.7	
Grade					0.473
Lower-division	266	58.1	364	60.3	
Upper-division	192	41.9	240	39.7	
Residential status					0.023^*^
With relatives	167	36.5	217	35.9	
On-Campus	127	27.7	210	34.8	
Off-campus	164	35.8	177	29.3	
Monthly disposable income					0.067
≤ 10,000 TWD	255	55.7	346	57.3	
10,001–15,000 TWD	103	22.5	158	26.2	
≥ 15,001 TWD	100	21.8	100	16.6	

*TWD* New Taiwan Dollar, ^a^ Differences based on chi-squared test, ^***^ *p* < 0.001, ^*^ *p* < 0.05

Please refer to Table 2 for the distribution of health-related information of university students by major in this study. Chi-squared analysis found that BMI (χ^2^ = 8.319, *p* = 0.016) and daily sleep duration (χ^2^ = 6.780, *p* = 0.034) significantly differed among the majors. More students with health-related majors (66.2%) than non-health-related majors (57.5%) had healthy weights (BMI = 18.5–23.9). More health-related students (57.6%) than non-health-related students (51.3%) slept seven hours or more per day.

**Table 2:** Health-related information of university students by major

Variables	Health-related majors (*n* = 458)		Non-health-related majors (*n* = 604)		*p*-value^a^
	*N*	%	*N*	%	
BMI (Kg/m^2^)					0.016*
< 18.5	69	15.1	115	19.0	
18.5–23.9	303	66.2	347	57.5	
≥ 24.0	86	18.8	142	23.5	
Daily sleep duration					0.034*
< 4 h	17	3.7	40	6.6	
4–6.9 h	177	38.6	254	42.1	
≥ 7 h	264	57.6	310	51.3	
Daily 3C usage duration					0.058
< 3 h	59	12.9	67	11.1	
3–5.9 h	138	30.1	224	37.1	
≥ 6 h	261	57.0	313	51.8	
Weekly physical activity duration					0.534
≤ 75 min	215	46.9	263	43.5	
76–150 min	142	31.0	202	33.4	
≥ 151 min	101	22.1	139	23.0	
Weekly dine-outs number					0.155
None	10	2.2	10	1.7	
1–14 times	233	50.9	275	45.5	
≥ 15 times	215	46.9	319	52.8	
Weekly night snacks number					0.248
None	94	20.5	134	22.2	
1–3 times	186	40.6	215	35.6	
≥ 4 times	178	38.9	255	42.2	

*BMI* Body Mass Index, *3C* Computer, Communication, and Consumer electronics, ^a^ Differences based on chi-squared test, ^*^ *p* < 0.05

### Perceived health status, health conception, and health-promoting lifestyle of different majors

See Table 3 for the PHS, HC, and HPLP scores for students from different majors in this study. Regarding PHS, no significant difference was observed between the mean scores for students with health-related (3.35 ± 0.95) and non-health-related (3.25 ± 0.94) majors.

In terms of HC (Table 3), the mean overall scores for students with health-related and non-health-related majors were 3.93 ± 0.60 and 3.81 ± 0.62, respectively, with the highest scores observed for adaptive dimension (4.10 ± 0.70, 3.95 ± 0.71), followed by functional/role (4.03 ± 0.68, 3.90 ± 0.71), eudaimonistic (3.88 ± 0.74, 3.80 ± 0.76), and clinical (3.58 ± 0.97, 3.49 ± 0.96) dimensions. The overall HC score (*t* = 3.173, *p* = 0.002), as well as the scores for functional/role (*t* = 3.182, *p* = 0.002) and adaptive (*t* = 3.511, *p* ˂ 0.001) dimensions of health-related students were significantly higher than those of non-health-related students.

Regarding the HPLP (Table 3), the mean overall score for health-related students was 3.39 ± 0.67; the highest score was observed for self-actualization dimension (3.61 ± 0.82), followed by interpersonal support (3.60 ± 0.78), stress management (3.50 ± 0.82), nutrition (3.31 ± 0.83), health responsibility (3.17 ± 0.89), and exercise (3.13 ± 0.92) dimensions. The mean overall score for non-health-related majors was 3.30 ± 0.69, with the ranking of each dimension being slightly different from that of health-related majors. The highest score was observed for interpersonal support dimension (3.57 ± 0.77), followed by self-actualization (3.53 ± 0.81), stress management (3.47 ± 0.77), nutrition (3.19 ± 0.87), exercise (3.06 ± 0.94), and health responsibility (3.00 ± 0.96) dimensions. Moreover, the overall HPLP score (*t* = 2.054, *p* = 0.040), as well as the scores for health responsibility (*t* = 2.947, *p* = 0.003) and nutrition (*t* = 2.176, *p* = 0.030) dimensions of health-related students were significantly higher than those of non-health-related students.

**Table 3:** PHS, HC, and HPLP of university students by major

Variables	Health-related majors (*n* = 458)		Non-health-related majors (*n* = 604)		*p*-value^a^
	Mean ± *SD*	Rank	Mean ± *SD*	Rank	
PHS	3.35 ± 0.95		3.25 ± 0.94		0.106
HC	3.93 ± 0.60		3.81 ± 0.62		0.002^**^
Functional/role	4.03 ± 0.68	2	3.90 ± 0.71	2	0.002^**^
Adaptive	4.10 ± 0.70	1	3.95 ± 0.71	1	<0.001^***^
Clinical	3.58 ± 0.97	4	3.49 ± 0.96	4	0.136
Eudaimonistic	3.88 ± 0.74	3	3.80 ± 0.76	3	0.075
HPLP	3.39 ± 0.67		3.30 ± 0.69		0.040^*^
Self-actualization	3.61 ± 0.82	1	3.53 ± 0.81	2	0.083
Health responsibility	3.17 ± 0.89	5	3.00 ± 0.96	6	0.003^**^
Exercise	3.13 ± 0.92	6	3.06 ± 0.94	5	0.179
Nutrition	3.31 ± 0.83	4	3.19 ± 0.87	4	0.030^*^
Interpersonal support	3.60 ± 0.78	2	3.57 ± 0.77	1	0.512
Stress management	3.50 ± 0.82	3	3.47 ± 0.77	3	0.436

*PHS* Perceived Health Status, *HC* Health Conception, *HPLP* Health-Promoting Lifestyle Profile, *SD* Standard Deviation, ^a^ Differences based on independent samples t-test, ^***^ *p* < 0.001, ^**^ *p* < 0.01, ^*^ *p* < 0.05

### Relationship between personal factors and perceived health status, health conception, and health-promoting lifestyle for students with different majors

#### Health-related majors

See Table 4 for the differences in PHS, HC, and HPLP scores stratified by personal factors among students with health-related majors. The PHS scores were significantly different for the different residential status (*F* = 4.924, *p* = 0.008), BMI (*F* = 4.186, *p* = 0.016), and daily sleep duration (*F* = 23.073, *p* ˂ 0.001). Post-hoc analysis showed that the PHS scores for students living in on-campus dormitories were significantly higher than those of students renting off-campus. Students who were underweight scored significantly higher than those who were overweight/obese. Participants who slept seven hours or more per day scored significantly higher than those who slept 4–6.9 h per day, while those who slept 4–6.9 h scored significantly higher than those who slept less than four hours daily.

**Table 4:** Relationship between personal factors and PHS, HC, and HPLP for university students with different majors

Variables	Health-related majors (*n* = 458)			Non-health-related majors (*n* = 604)		
	PHS	HC	HPLP	PHS	HC	HPLP
	Mean ± *SD*	Mean ± *SD*	Mean ± *SD*	Mean ± *SD*	Mean ± *SD*	Mean ± *SD*
Gender						
Male	3.39 ± 0.91	3.89 ± 0.64	3.49 ± 0.65	3.35 ± 0.91	3.83 ± 0.65	3.41 ± 0.76
Female	3.33 ± 0.96	3.95 ± 0.59	3.34 ± 0.67	3.16 ± 0.96	3.79 ± 0.59	3.20 ± 0.59
*p*-value^a^	0.467	0.366	0.022^*^	0.015^*^	0.429	<0.001^***^
Grade						
Lower-division	3.36 ± 0.98	3.91 ± 0.59	3.36 ± 0.67	3.24 ± 0.93	3.77 ± 0.63	3.29 ± 0.68
Upper-division	3.33 ± 0.90	3.95 ± 0.63	3.43 ± 0.67	3.28 ± 0.96	3.87 ± 0.60	3.32 ± 0.70
*p*-value^a^	0.689	0.411	0.256	0.645	0.051	0.512
Residential status						
With relatives	3.40 ± 0.89 ^ab^	3.81 ± 0.63 ^b^	3.35 ± 0.66 ^ab^	3.28 ± 0.89	3.81 ± 0.68	3.32 ± 0.72
On-Campus	3.51 ± 0.93 ^a^	4.04 ± 0.60 ^a^	3.54 ± 0.69 ^a^	3.29 ± 0.94	3.77 ± 0.58	3.27 ± 0.62
Off-campus	3.17 ± 0.99 ^b^	3.97 ± 0.56 ^ab^	3.31 ± 0.65 ^b^	3.18 ± 1.00	3.86 ± 0.60	3.32 ± 0.73
*p*-value^b^	0.008^**^	0.003^**^	0.009^**^	0.458	0.345	0.699
Monthly disposable income						
≤ 10,000 TWD	3.29 ± 1.00	4.00 ± 0.63	3.41 ± 0.73	3.18 ± 0.94 ^b^	3.81 ± 0.62	3.22 ± 0.69^b^
10,001–15,000 TWD	3.29 ± 0.88	3.84 ± 0.55	3.32 ± 0.57	3.27 ± 0.89 ^ab^	3.75 ± 0.59	3.31 ± 0.60^b^
≥ 15,001 TWD	3.55 ± 0.86	3.83 ± 0.58	3.39 ± 0.61	3.48 ± 0.98 ^a^	3.90 ± 0.65	3.57 ± 0.73^a^
*p*-value^b^	0.050^N.S.^	0.014^N.S.^	0.509	0.017^*^	0.159	<0.001^***^
BMI (Kg/m^2^)						
< 18.5	3.63 ± 0.88 ^a^	3.90 ± 0.62	3.49 ± 0.67	3.08 ± 0.98 ^b^	3.84 ± 0.56	3.24 ± 0.67
18.5–23.9	3.32 ± 0.94 ^ab^	3.94 ± 0.58	3.38 ± 0.66	3.34 ± 0.94 ^a^	3.85 ± 0.60	3.35 ± 0.67
≥ 24.0	3.22 ± 0.98 ^b^	3.89 ± 0.68	3.34 ± 0.70	3.19 ± 0.88 ^ab^	3.68 ± 0.70	3.22 ± 0.75
*p*-value^b^	0.016^*^	0.766	0.385	0.025^*^	0.025^N.S.^	0.089
Daily sleep duration						
< 4 h	2.53 ± 0.82 ^c^	3.84 ± 0.76	3.09 ± 0.73 ^b^	2.88 ± 1.10 ^b^	3.39 ± 0.74^b^	2.94 ± 0.90^b^
4–6.9 h	3.08 ± 0.96 ^b^	3.84 ± 0.59	3.28 ± 0.66 ^ab^	3.13 ± 0.90 ^ab^	3.80 ± 0.58^a^	3.24 ± 0.61^a^
≥ 7 h	3.58 ± 0.87 ^a^	3.99 ± 0.60	3.48 ± 0.66 ^a^	3.40 ± 0.92 ^a^	3.87 ± 0.62^a^	3.40 ± 0.70^a^
*p*-value^b^	<0.001^***^	0.032^N.S.^	0.001^**^	<0.001^***^	<0.001^***^	<0.001^***^
Daily 3C usage duration						
< 3 h	3.39 ± 0.89	3.84 ± 0.68	3.34 ± 0.72	3.13 ± 1.04	3.69 ± 0.74	3.30 ± 0.75
3–5.9 h	3.26 ± 0.97	3.92 ± 0.60	3.32 ± 0.73	3.31 ± 0.96	3.83 ± 0.64	3.34 ± 0.68
≥ 6 h	3.38 ± 0.95	3.95 ± 0.59	3.43 ± 0.62	3.24 ± 0.90	3.81 ± 0.58	3.28 ± 0.68
*p*-value^b^	0.467	0.403	0.244	0.379	0.276	0.636
Weekly physical activity duration						
≤ 75 min	3.27 ± 0.95	3.89 ± 0.64	3.23 ± 0.71 ^b^	3.08 ± 0.98 ^b^	3.77 ± 0.65 ^b^	3.16 ± 0.74^b^
76–150 min	3.45 ± 0.99	3.94 ± 0.58	3.52 ± 0.61 ^a^	3.29 ± 0.84 ^b^	3.78 ± 0.57 ^ab^	3.35 ± 0.56^a^
≥ 151 min	3.38 ± 0.86	3.98 ± 0.57	3.55 ± 0.58 ^a^	3.52 ± 0.94 ^a^	3.93 ± 0.63 ^a^	3.50 ± 0.72^a^
*p*-value^b^	0.177	0.456	<0.001^***^	<0.001^***^	0.034^*^	<0.001^***^
Weekly dine-outs number						
None	3.23 ± 0.79	3.50 ± 0.57 ^b^	3.32 ± 0.62	3.43 ± 1.14	3.09 ± 1.08^b^	3.38 ± 1.20
1–14 times	3.42 ± 0.88	3.91 ± 0.61 ^a^	3.41 ± 0.68	3.31 ± 0.93	3.83 ± 0.62^a^	3.37 ± 0.67
≥ 15 times	3.28 ± 1.02	3.97 ± 0.60 ^a^	3.37 ± 0.66	3.20 ± 0.94	3.81 ± 0.59^a^	3.24 ± 0.68
*p*-value^b^	0.273	0.044^*^	0.770	0.320	0.001^**^	0.060
Weekly night snacks number						
None	3.43 ± 0.96	4.01 ± 0.64	3.45 ± 0.68	3.20 ± 0.93	3.72 ± 0.68	3.28 ± 0.71
1–3 times	3.32 ± 0.95	3.92 ± 0.58	3.33 ± 0.66	3.23 ± 0.94	3.82 ± 0.60	3.28 ± 0.66
≥ 4 times	3.33 ± 0.94	3.89 ± 0.61	3.41 ± 0.67	3.30 ± 0.94	3.84 ± 0.60	3.33 ± 0.71
*p*-value^b^	0.627	0.332	0.335	0.596	0.146	0.628

*PHS* Perceived Health Status, *HC* Health Conception, *HPLP* Health-Promoting Lifestyle Profile, *SD* Standard Deviation, *TWD New* Taiwan Dollar, *BMI* Body Mass Index, *3C* Computer, Communication, and Consumer electronics, ^a^ Differences based on independent samples t-test, ^b^ Differences based on one-way ANOVA, ^***^ *p* < 0.001, ^**^ *p* < 0.01, ^*^ *p* < 0.05. Values with different superscript letters in variables with more than two categories indicate significant difference by Scheffé post-hoc test, while ^N.S.^ indicates there is no significant difference by post-hoc test.

Regarding the overall HC (Table 4), the scores for students with health-related majors significantly differed with residential status (*F* = 5.799, *p* = 0.003) and weekly dine-outs number (*F* = 3.153, *p* = 0.044). Post-hoc analysis showed that students living in on-campus dormitories scored significantly higher on HC than those living with relatives. Students who dined out also scored significantly higher.

Regarding the overall HPLP (Table 4), the scores for students with health-related majors significantly differed with gender (*t* = 2.302, *p* = 0.022), residential status (*F* = 4.804, *p* = 0.009), daily sleep duration (*F* = 6.617, *p* = 0.001), and weekly physical activity duration (*F* = 12.165, *p* ˂ 0.001). Male students had significantly higher HPLP scores. Students living in on-campus dormitories had significantly higher scores than those renting off-campus. Participants who slept seven hours or more per day scored significantly higher than those who slept less than four hours per day. Students who exercised more than 75 min per week also scored significantly higher.

#### Non-health-related majors

Significant differences were observed in the PHS scores for non-health-related students (Table 4) based on gender (*t* = 2.439, *p* = 0.015), monthly disposable income (*F* = 4.130, *p* = 0.017), BMI (*F* = 3.699, *p* = 0.025), daily sleep duration (*F* = 9.266, *p* ˂ 0.001), and weekly physical activity duration (*F* = 10.515, *p* ˂ 0.001). Male students had significantly higher PHS scores. Students with a monthly disposable income of more than 15,000 TWD had significantly higher scores than those with 10,000 TWD or less. Participants with a healthy weight scored significantly higher than those who were underweight. Students who slept seven hours or more per day scored significantly higher than those who slept less than four hours a day. Participants who exercised more than 150 min per week also scored significantly higher.

In terms of the overall HC (Table 4), significant differences were observed in the scores for students with non-health-related majors based on daily sleep duration (*F* = 10.912, *p* ˂ 0.001), weekly physical activity duration (*F* = 3.410, *p* = 0.034), and weekly dine-outs number (*F* = 6.916, *p* = 0.001). Post-hoc analysis showed that the HC scores were significantly higher for those who slept four hours or more per day. The scores for students who exercised more than 150 min per week were significantly higher than those who exercised 75 min or less. The significantly higher scores were also observed for participants who dined out weekly.

In terms of the overall HPLP (Table 4), the scores for non-health-related students were significantly different by gender (*t* = 3.811, *p* ˂ 0.001), monthly disposable income (*F* = 10.271, *p* ˂ 0.001), daily sleep duration (*F* = 10.024, *p* ˂ 0.001), and weekly physical activity duration (*F* = 12.276, *p* ˂ 0.001). Among them, significantly higher HPLP scores were observed for males, students with a monthly disposable income of more than 15,000 TWD, those who slept four hours or more a day, and those who exercised more than 75 min per week.

### Correlations between perceived health status, health conception, and health-promoting lifestyle in different majors

See Table 5 for the correlations between PHS, HC, and HPLP scores for students with different majors using the Pearson product-moment correlation analysis. Among health-related and non-health-related majors, significant positive correlations were observed between the PHS score and the overall HPLP score (*r* = 0.516, *p* < 0.001 and *r* = 0.457, *p* < 0.001), as well as the scores for each dimension of HPLP (*r* = 0.332–0.468, *p* < 0.001 and *r* = 0.314–0.408, *p* < 0.001). There were also significant positive correlations between the overall HC score and the overall HPLP score (*r* = 0.584, *p* < 0.001 and *r* = 0.576, *p* < 0.001), with significant positive correlations observed between each dimension of HC and each dimension of HPLP (*r* = 0.228–0.616, *p* < 0.001 and *r* = 0.235–0.524, *p* < 0.001).

**Table 5:** Pearson correlations between PHS, HC, and HPLP for university students with different majors

Variables	Health-related majors (*n* = 458)						Non-health-related majors (*n* = 604)					
	PHS	HC					PHS	HC				
		Overall	Functional/role	Adaptive	Clinical	Eudaimonistic		Overall	Functional/role	Adaptive	Clinical	Eudaimonistic
	*r*	*r*	*r*	*r*	*r*	*r*	*r*	*r*	*r*	*r*	*r*	*r*
HPLP												
Overall	0.516^***^	0.584^***^	0.484^***^	0.454^***^	0.408^***^	0.531^***^	0.457^***^	0.576^***^	0.510^***^	0.438^***^	0.389^***^	0.529^***^
Self-actualization	0.441^***^	0.654^***^	0.560^***^	0.561^***^	0.368^***^	0.616^***^	0.398^***^	0.561^***^	0.524^***^	0.484^***^	0.288^***^	0.519^***^
Health responsibility	0.402^***^	0.474^***^	0.348^***^	0.316^***^	0.410^***^	0.452^***^	0.370^***^	0.392^***^	0.335^***^	0.239^***^	0.315^***^	0.389^***^
Exercise	0.332^***^	0.341^***^	0.261^***^	0.228^***^	0.312^***^	0.289^***^	0.362^***^	0.365^***^	0.321^***^	0.235^***^	0.293^***^	0.335^***^
Nutrition	0.468^***^	0.358^***^	0.301^***^	0.251^***^	0.267^***^	0.331^***^	0.365^***^	0.434^***^	0.402^***^	0.313^***^	0.305^***^	0.382^***^
Interpersonal support	0.404^***^	0.492^***^	0.422^***^	0.402^***^	0.311^***^	0.447^***^	0.314^***^	0.525^***^	0.465^***^	0.449^***^	0.316^***^	0.464^***^
Stress management	0.417^***^	0.480^***^	0.433^***^	0.425^***^	0.269^***^	0.408^***^	0.408^***^	0.557^***^	0.461^***^	0.458^***^	0.378^***^	0.506^***^

*PHS* Perceived Health Status, *HC* Health Conception, *HPLP* Health-Promoting Lifestyle Profile, *r* Correlation coefficient, ^***^ *p* < 0.001

### Influencing factors for the prediction of health-promoting lifestyle in different majors

First, the predictors of overall HPLP score for all university students (Additional file 1) were analyzed by stepwise multiple regression. Gender, weekly physical activity duration, weekly dine-outs number, PHS, and HC were predictors of overall HPLP, which explained 46.0% of the variance (adjusted R^2^ = 0.460). The HPLP score of males was higher than that of females by 0.109 points. Compared with students who exercised 75 min or less per week, the HPLP scores of those who exercised 76–150 min and more than 150 min were increased by 0.176 and 0.190 points, respectively. Compared with students who dined out 15 times or more per week, the HPLP scores of those who did not dine out and dined out 1–14 times were increased by 0.295 and 0.091 points, respectively. Every increase in PHS score and scores for functional/role, clinical, and eudaimonistic dimensions of HC increased the HPLP score by 0.199, 0.219, 0.081, and 0.225 points, respectively. There was no collinearity between the various independent variables (tolerance = 0.470–0.967; VIF = 1.034–2.126). See Table 6 for the regression analysis results by major. Among health-related majors, gender, weekly physical activity duration, PHS, and HC were predictors of HPLP, which explained 48.1% of the variance (adjusted R^2^ = 0.481). The HPLP score of males was higher than that of females by 0.100 points. Compared with students who exercised 75 min or less per week, the HPLP scores of those who exercised 76–150 min and more than 150 min were increased by 0.217 and 0.238 points, respectively. Every increase in PHS score and scores for functional/role, clinical, and eudaimonistic dimensions of HC increased the HPLP score for students with health-related majors by 0.238, 0.155, 0.080, and 0.249 points, respectively. There was no collinearity between the various independent variables (tolerance = 0.475–0.936; VIF = 1.068–2.105). Among non-health-related majors, gender, monthly disposable income, weekly dine-outs number, PHS, and HC were predictors of HPLP, which explained 44.3% of the variance (adjusted R^2^ = 0.443). The HPLP score of males was higher than that of females by 0.132 points. Compared with students with a monthly disposable income of more than 15,000 TWD, the HPLP scores of those with a monthly disposable income of less than 10,001 TWD and 10,001–15,000 TWD were decreased by 0.223 and 0.138 points, respectively. Compared with students who dined out 15 times or more per week, the HPLP scores of those who did not dine out and dined out 1–14 times were increased by 0.443 and 0.120 points, respectively. Every increase in PHS score and scores for functional/role, clinical, and eudaimonistic dimensions of HC increased the HPLP score for students with non-health-related majors by 0.178, 0.257, 0.068, and 0.212 points, respectively. There was no collinearity between the various independent variables (tolerance = 0.465–0.961; VIF = 1.040–2.151).

**Table 6:** Multiple regression analysis of overall HPLP for university students with different majors

Predictors	Health-related majors (*n* = 458)			Non-health-related majors (*n* = 604)		
	B	β	*p*-value	B	β	*p*-value
(Constant)	0.558		<0.001^***^	0.716		<0.001^***^
Socio-demographic characteristics						
Male	0.100	0.070	0.046^*^	0.132	0.096	0.002^**^
Monthly income ≤ 10,000 TWD				-0.223	-0.161	<0.001^***^
Monthly income 10,001–15,000 TWD				-0.138	-0.088	0.039^*^
Health-related information						
Weekly physical activity 76–150 min	0.217	0.150	<0.001^***^			
Weekly physical activity ≥ 151 min	0.238	0.148	<0.001^***^			
Weekly none dine-outs				0.443	0.082	0.008^**^
Weekly 1–14 times dine-outs				0.120	0.087	0.005^**^
PHS	0.238	0.337	<0.001^***^	0.178	0.243	<0.001^***^
HC						
Functional/role	0.155	0.158	<0.001^***^	0.257	0.264	<0.001^***^
Clinical	0.080	0.117	0.005^**^	0.068	0.095	0.010^*^
Eudaimonistic	0.249	0.274	<0.001^***^	0.212	0.234	<0.001^***^
Adjusted *R*^2^	0.481			0.443		
*p*-value	<0.001^***^			<0.001^***^		

*HPLP* Health-Promoting Lifestyle Profile, *B* Estimate, β Standardized Estimate, *TWD* New Taiwan Dollar, *PHS* Perceived Health Status, *HC* Health Conception, *R* Correlation coefficient, *R*^2^ Coefficient of determination, ^***^ *p* < 0.001, ^**^ *p* < 0.01, ^*^ *p* < 0.05

## Discussion

There were slightly more females in the overall sample in this study. There were only 1.4% more female than male students with non-health-related majors, however, there were 35.8% more female students with health-related majors. This result was similar to the gender statistics of the discipline clusters in universities in Taiwan. Female students outnumbered male students by 46.1%, 14.7%, and 16.0% for medicine and health, hospitality/tourism/personal service, and life sciences clusters, respectively. Only the agriculture cluster had slightly more males than females by 5.4% [31]. More than half of the participants with health-related majors in this study were medical and health students, with only a few participants majoring in agriculture, which made it even more obvious that there were more female students than male students. Studies have shown that university students chose their majors due to personal characteristics or social expectations [16, 32, 33], and families, schools, and workplaces jointly constructed or strengthened the gender inequality of students in their choices of majors [34]. In addition, more non-health-related students were living in dormitories on campus in this study, while more health-related students rented apartments off campus. To facilitate management, school dormitories have more restrictions on the behavior and schedule of students than off-campus accommodations do. This study also found that health-related students had better scores for adaptive dimension of HC and health responsibility dimension of HPLP. This could be explained by the fact that more students with health-related majors lived with their relatives or off-campus accommodations and had more freedom in their lifestyles and schedules. Therefore, after acquiring accurate and sufficient health knowledge, they were able to adapt to the environment and were more confident in being responsible for their own health.

A regular daily routine, balanced diet, and adequate physical activity are the keys to maintaining a healthy body weight. In this study, more health-related students had a healthy weight than non-health-related students, which was speculated to be related to the type of courses planned based on their majors. Studies have shown that students majoring in health-related disciplines were typically more exposed to health-related courses [7, 33], and students with exposure to health-related courses tended to exhibit better dietary behaviors [8, 9], exercise behaviors [35], and other health-promoting behaviors [17, 18], which may have made it easier for them to maintain a healthy weight. This finding also was consistent with the higher scores for overall HC, overall HPLP, as well as health responsibility and nutrition dimensions of HPLP of health-related students in this study. Furthermore, recent studies have shown the relationships between healthy body weight, adequate sleep duration, and high sleep efficiency [36], which may also explain the finding that more health-related students had adequate sleep than non-health-related students in this study.

The highest HC scores for university students in this study were observed for the adaptive dimension regardless of the major, followed by functional/role performance, eudaimonistic, and clinical dimensions, which was similar to the findings of other studies [29, 37]. Compared with recent literature, this study further found that the overall HC score, as well as the scores for adaptive and functional/role dimensions of health-related students, were higher than those of non-health-related students. It was speculated that the definition of health may vary with individuals’ learning content or social roles. University students are receiving higher education and preparing for employment, and they must be more concerned about their adaptability and performance. Therefore, this study suggested that health-related students who have been exposed to more health-related courses and would become health providers in the future would agree more on the fact that health was the ability to flexibly adapt to the changes in the external environment, and was being able to fully perform the desired social role.

In terms of HPLP of different majors, the rankings of various dimensions slightly differed, however, the higher scores were observed for self-actualization and interpersonal support, while lower scores were observed for nutrition, exercise, and health responsibility, which was consistent with most recent studies [8, 11, 16–20]. This finding also was compatible with that regardless of the major in this study, above 40% of university students exercise 75 min or less per week, nearly half of all students dine out two times or more a day, and above 70% eat night snacks. Studies have pointed out that the lack of information or e-health literacy often contributed to malnutrition and poor dietary behaviors of modern university students [24, 38], while the lack of time, insufficient facilities, or weak willpower were the excuses that students often used to avoid exercise [20, 38, 39]. Moreover, university students gradually become independent of their families and would receive less care related to their health from their relatives [8]. Those students are usually still very healthy and have not yet realized that health responsibility is a necessary and important concept [20], leading to unsatisfactory behaviors such as nutrition, exercise, and health responsibility. In addition, the results of this study showed that the overall HPLP score and the scores for nutrition and health responsibility dimensions of health-related students were higher than those of non-health-related students. In the health responsibility dimension, this finding was compatible with the results that more health-related students, than non-health-related students, had a healthy weight and adequate sleep in this study. Although the health-related students had fewer dine-outs and night snacks than non-health-related students in this study, the difference was not significant. This may be because the nutrition dimension of HPLP focuses on the selection of food types and not the source and timing of diets. Recent studies have also found that students majoring in medicine, sports, and health had better overall HPLP [10, 18, 23], while better scores in the health responsibility dimension were observed for students majoring in health and natural sciences [7, 19]. Moreover, medical students had higher scores for nutrition dimension [8]. These behavioral differences by discipline are considered to be related to the interests and characteristics of the students when choosing their departments, as well as the content of subsequent courses arranged by each department [35, 40].

In this study, stepwise multiple regression analysis was performed to identify the predictors of overall HPLP in university students. For all students and students with different majors, the PHS and HC for cognitive-perceptual factors, as well as gender for modifying factors were all important indicators for HPLP. Females, students with low PHS scores, and those with low scores for functional/role, clinical, and eudaimonistic dimensions of HC had a relatively negative HPLP, making them the target groups for urgent attention and correction of health-promoting behaviors. This finding was also consistent with the significant gender differences in HPLP among health-related and non-health-related students. Moreover, this finding echoed the positive correlations between HPLP and both PHS and HC. Recent studies have demonstrated that favorable PHS and HC may also predict good HPLP of the individual [21, 27, 29]. In addition, most studies have indicated that the genders of university students may be used to predict their HPLP. Some scholars have suggested that females have better HPLP [8, 29], while some scholars believe that males have better HPLP [21, 41]. Some studies have suggested that gender is not a predictor of HPLP [18]. It is speculated that gender would have different predictive effects on participants in different regions. In this study, the author believed that gender may still serve as a valuable reference for university students in Taiwan.

In this study, there were other noteworthy modifying factors for HPLP for different majors, in addition to factors such as gender, PHS, and HC. For health-related majors, the HPLP for students was also affected by weekly physical activity duration, with those who exercised 75 min or less per week demonstrating a negative HPLP. Maintaining regular exercise habits has been known to improve various health-promoting behaviors and is a significant factor affecting the HPLP of university students [18, 22, 42]. Based on the World Health Organization, the Health Promotion Administration of the Ministry of Health and Welfare of Taiwan recommended that adults should perform at least 150 min of moderate physical activity or 75 min of strenuous physical activity per week cumulatively [43]. Although this study did not consider exercise intensity, under 55% of health-related students exercised more than 75 min weekly, and only 22.1% exercised more than 150 min weekly. Furthermore, 57% of health-related students used 3C products for six hours or more a day. Recent studies have also found that the use of 3C products and the dominance of the Internet may make university students tend to be sedentary [8, 16]. The author considered that electronicalization of learning and lifestyle in today’s world increases the convenience of knowledge acquisition, but decreases the opportunities for physical activity. In particular, health-related students who are already programmed with many health courses should be offered more opportunities for classroom- or campus-related activities to cultivate health-promoting lifestyles. In addition, the PHS, HC, and HPLP for health-related students in this study significantly differed with residential status and sleep duration. However, the multiple regression analysis showed that residential status and sleep duration were excluded from the predictors. It was speculated that the influence of these two factors on HPLP had been explained by cognitive-perceptual factors.

For non-health-related majors, the HPLP for students was also affected by monthly disposable income and weekly dine-outs number, in addition to factors such as gender, PHS, and HC. Students with a monthly disposable income of 15,000 TWD or less and those who dined out 15 times or more a week required attention for health-promoting behaviors. Studies have shown that university students with higher incomes generally exhibited better HPLP [44]. Moreover, family or personal socioeconomic status was a significant factor affecting HPLP [41, 45]. In addition, the Nutrition and Health Survey in Taiwan from 2017 to 2020 showed that adults aged 19–44 years often ate out because of school or work. Among them, 2.7%, 51.8%, and 45.5% of people ate out 0 times, less than 14 times, and 14 times or more a week, respectively [46]. The frequency of eating out gradually decreased with age, and has been associated with potential concerns such as the choices of food types, the quality of oil and salt, and the hygiene and safety of food preparation [44, 46]. Studies have also confirmed that eating out (especially fast food) was likely to negatively affect the nutritional status, body image, and health-promoting behaviors of individuals [38, 44]. In this study, 52.8% of non-health-related students dined out 15 times or more a week, which is higher than the overall dine-out frequency in Taiwanese adults. Furthermore, the result also showed approximately 25% of non-health-related students were overweight or obese. It was speculated that university students who do not pay enough attention to their diet dine out more frequently to accommodate their peers, their busy lifestyle, or succumb to flashy advertisements. Therefore, especially for non-health-related students who are exposed to less health courses, it is important to acquire accurate knowledge about nutrition and food selection as early as possible. In addition, the PHS, HC, and HPLP for students with non-health-related majors significantly differed with sleep and physical activity durations in this study, but these two factors were excluded from the predictors of the multiple regression analysis. It was speculated that the effects of these two factors on HPLP may have also been explained by cognitive-perceptual factors.

There are limitations to this study that may serve as references for related research in the future. Firstly, due to the cross-sectional design, this study could not explain the real changes in HPLP over time. Therefore, further elucidation is required to determine the causal relationship between the variables. Secondly, the self-administered questionnaire may lead to concerns about the accuracy of the responses due to memory error, social desirability bias, and environmental influence of the participants. In this study, an anonymous questionnaire was adopted with an increase in sample size to minimize the effects of bias. In addition, the personality traits, interpersonal relationships, religious beliefs, cultural backgrounds, and health risk behaviors of participants were not included as variables in this study, which is suggested to explore in future research. Furthermore, the scope of this study did not include the outlying islands of Taiwan. Considering that university students on the outlying islands may have different lifestyles from those on the main island, corresponding health education strategies should be independently discussed and developed. Despite the above research limitations, this study provided unprecedented progress on the differences in the HPLP for Taiwanese university students in different majors, as well as proposed specific countermeasures.

## Conclusions

Although higher education training corresponds to the requirements of disciplines and majors, health should not differ with discipline or major. In this study, it was found that health-promoting behaviors such as nutrition, exercise, and health responsibility of health-related and non-health-related students in Taiwan had to be improved. However, the HC and HPLP for non-health-related students were more worrying than those of health-related students. The author suggested that universities should help students establish a positive HC and strengthen their awareness of balanced nutrition, regular exercise, and paying attention to their health. In particular, non-health-related students should be prioritized in knowledge promotion. In addition, there were differences in the predictors of HPLP between health-related and non-health-related majors in Taiwan. Universities should identify targets with poor HPLP who require urgent and prioritized intervention according to their professional fields, including health-related students with inadequate physical activity, non-health-related students with low disposable income or excessive dining out, female students in all majors, as well as students with poor PHS score and poor scores for functional/role, clinical, and eudaimonistic dimensions of HC to provide appropriate and effective health promotion education and programs. For example, the design of an on-campus sports support program for health-related students with insufficient physical activity is recommended to focus on teaching the importance of sports, the construction of sports facilities, and the planning of sports courses. A nutritional support plan may also be designed for non-health-related students who frequently eat out to emphasize the relationship between diet and health. Healthy eating patterns should be facilitated by the campus cafeteria, and a plan should be dedicated to connecting the restaurants around the campus to create a friendly environment where students can dine out safely. The author considered that it is important and necessary for university students in Taiwan to “live healthy” by “learning and practicing healthy.”

## Supplementary Information

**Additional file 1.** Multiple regression analysis of overall HPLP for all university students (*n* = 1062). The predictors of overall HPLP score for all university students were analyzed by stepwise multiple regression. Among them, a significantly better HPLP score was observed for males, and students who exercised more than 75 min per week, those dined out less than 15 times per week, as well as those with higher PHS scores and scores for functional/role, clinical, and eudaimonistic dimensions of HC, which explained 46.0% of the variance (adjusted R^2^= 0.460).

## Data Availability

The datasets generated and/or analyzed during the current study are not publicly available due to confidentiality in the informed consent. However, the datasets are available from the corresponding author upon reasonable request.

## Abbreviations

3C: Computer, Communication, and Consumer electronics
BMI: Body Mass Index
HC: Health Conception
HPL: Health-Promoting Lifestyle
HPLP: Health-Promoting Lifestyle Profile
MOE: Ministry of Education
PHS: Perceived Health Status
