# Supplementary material for: Health-promoting lifestyle and its predictors among health-related and non-health-related university students in Taiwan: a cross-sectional quantitative study
Source: BMC Public Health. 2023 May 5;23:827. doi: 10.1186/s12889-023-15760-2 (PMC10161567; doi:10.1186/s12889-023-15760-2)
Supplement: Supplementary file 1 — Additional file 1. Multiple regression analysis of overall HPLP for all university students (n = 1062). The predictors of overall HPLP score for all university students were analyzed by stepwise multiple regression. Among them, a significantly better HPLP score was observed for males, and students who exercised more than 75 min per week, those dined out less than 15 times per week, as well as those with higher PHS scores and scores for functional/role, clinical, and eudaimonistic dimensions of HC, which explained 46.0% of the variance (adjusted R2= 0.460). [file 12889_2023_15760_MOESM1_ESM.docx]

Name and Format: Additional file 1.docx

Title: Multiple regression analysis of overall HPLP for all university students (n = 1062)

Description: The predictors of overall HPLP score for all university students were analyzed by stepwise multiple regression. Among them, a significantly better HPLP score was observed for males, and students who exercised more than 75 minutes per week, those dined out less than 15 times per week, as well as those with higher PHS scores and scores for functional/role, clinical, and eudaimonistic dimensions of HC, which explained 46.0% of the variance (adjusted R^2^ = 0.460).

**Additional file 1** Multiple regression analysis of overall HPLP for all university students (*n* = 1062)

| Predictors | B | β | | *p*-value |  |
| --- | --- | --- | --- | --- | --- |
| (Constant) | 0.477 |  | | <0.001 | ^***^ |
| Socio-demographic characteristics |  |  | |  |  |
| Male | 0.109 | 0.079 | | <0.001 | ^***^ |
| Health-related information |  |  | |  |  |
| Weekly physical activity 76–150 min | 0.176 | 0.121 | | <0.001 | ^***^ |
| Weekly physical activity ≥ 151 min | 0.190 | 0.117 | | <0.001 | ^***^ |
| Weekly none dine-outs | 0.295 | 0.059 | | 0.011 | ^*^ |
| Weekly 1–14 times dine-outs | 0.091 | 0.067 | | 0.004 | ^**^ |
| PHS | 0.199 | 0.275 | | <0.001 | ^***^ |
| HC |  |  | |  |  |
| Functional/role | 0.219 | 0.225 | | <0.001 | ^***^ |
| Clinical | 0.081 | 0.114 | | <0.001 | ^***^ |
| Eudaimonistic | 0.225 | 0.248 | | <0.001 | ^***^ |
| Adjusted *R*^2^ | 0.460 | |  | | |
| *p*-value | <0.001 | | ^***^ | | |

HPLP Health-Promoting Lifestyle Profile, B Estimate, β Standardized Estimate, PHS Perceived Health Status, HC Health Conception, *R* Correlation coefficient, *R*^2^ Coefficient of determination, ^***^ *p* < 0.001, ^**^ *p* < 0.01, ^*^ *p* < 0.05
